# Ovarian Collision Tumor in a Pediatric Patient: A Mature Teratoma Associated with a Combined Tumor Containing a Mucinous Cystadenocarcinoma Component

**DOI:** 10.3390/jcm14186387

**Published:** 2025-09-10

**Authors:** Dorotea Keretić, Ivan Petračić, Silvija Mašić Binder, Monika Ulamec, Andrea Plavec Živko, Jasminka Stepan Giljević, Aleksandra Bonevski, Dubravko Habek, Marko Bašković

**Affiliations:** 1 Department of Pediatric Surgery, Children’s Hospital Zagreb, Ulica Vjekoslava Klaića 16, 10000 Zagreb, Croatia; 2 School of Medicine, University of Zagreb, Šalata 3, 10000 Zagreb, Croatia; 3 Ljudevit Jurak Clinical Department of Pathology and Cytology, University Clinical Hospital Center Sestre Milosrdnice, Vinogradska cesta 29, 10000 Zagreb, Croatia; 4 Scientific Centre of Excellence for Reproductive and Regenerative Medicine, School of Medicine, University of Zagreb, Šalata 3, 10000 Zagreb, Croatia; 5 Department of Pediatrics, Polyclinic for Pediatric and Adolescent Gynecology and Reproductive Health, Children’s Hospital Zagreb, Ulica Vjekoslava Klaića 16, 10000 Zagreb, Croatia; 6 Department of Pediatric Oncology and Hematology, Children’s Hospital Zagreb, Ulica Vjekoslava Klaića 16, 10000 Zagreb, Croatia; 7 Department of Obstetrics and Gynecology, Clinical Hospital Merkur, Zajčeva ulica 19, 10000 Zagreb, Croatia; 8 School of Medicine, Catholic University of Croatia, Ilica 242, 10000 Zagreb, Croatia; 9 Croatian Academy of Medical Sciences, Kaptol 15, 10000 Zagreb, Croatia

**Keywords:** cystadenoma, cystadenocarcinoma, borderline tumor, mature teratoma, collision tumor, composed tumor, ovary, children, adolescent, surgery

## Abstract

**Background**: Collision tumors, especially in the ovary, are a rare phenomenon where two distinct types of tumors develop adjacent to each other within the same organ but remain separate histologically. We present a case of the first collision ovarian tumor in a 14-year-old girl consisting of a combined tumor and a mature teratoma. **Case Report**: A 14-year-old girl presented with abdominal swelling for the past three months, without other symptoms. Ultrasound (US) examination of the abdomen revealed a large cystic mass with multiple septa, filling the entire abdomen from the diaphragm to the pelvis. Magnetic resonance imaging (MRI) showed an intraperitoneal mass, inseparable from the right ovary, measuring 22 cm × 13 cm × 30 cm. Serum tumor markers were within normal limits. The tumor mass was completely extirpated along with the fallopian tube. Histological and immunohistochemical analysis determined that it was a mucinous cystadenocarcinoma, characterized by a transition pattern from benign and borderline components to an adenocarcinoma component with a smaller mature teratoma. Six-month follow-up revealed no recurrence or postoperative complications. **Conclusions**: As the first documented case, this case provides valuable insights into pediatric ovarian neoplasms, guiding future diagnostic and therapeutic approaches.

## 1. Introduction

Ovarian neoplasms are rare in children and adolescents, with a reported annual incidence of 2.6 cases per 100,000 girls. An extremely small number of ovarian neoplasms in this age group are malignant. When they do occur, they are often germ cell tumors like yolk sac tumors, dysgerminomas, or immature teratomas [1,2,3]. Due to the small number of reported cases in children and adolescents, the diagnosis and treatment present many challenges [4,5].

Mature ovarian teratomas, also known as dermoid cysts, are the most common ovarian germ cell tumors in children and adolescents [6]. They are typically benign and composed of well-differentiated tissues derived from all three germ layers: ectoderm, mesoderm, and endoderm [7]. They are usually asymptomatic and discovered incidentally, and when symptomatic, they often present with abdominal or pelvic pain, or rarely, signs of ovarian torsion or rupture [2,6,8]. Malignant transformation is rare, but long-term follow-up is recommended if any suspicious features are present [9].

Mucinous cystadenocarcinoma of the ovary is an extremely rare malignant ovarian tumor in children and adolescents. It occurs predominantly in women of reproductive age [10]. It is characterized by mucin-producing epithelial cells with malignant features, and usually arises from benign or borderline mucinous cystadenomas, progressing through a borderline stage to invasive carcinoma [11]. Serum tumor markers such as CA-125 may be elevated but are not specific, and definitive diagnosis requires histopathological examination after surgical removal, with a fertility-sparing surgical approach (e.g., unilateral salpingo-oophorectomy) being preferred. Chemotherapy is considered in advanced or metastatic cases [12,13,14].

Collision tumors are a fascinating and particularly rare phenomenon in pathology. They arise when two distinct tumors, originating from different cell lineages or tissue types, develop independently alongside each other within the same organ or anatomical site and eventually come into contact, maintaining distinct histological borders without intermingling. Recognition of collision tumors is important because they can affect treatment planning and prognosis, especially if one component is malignant [15,16,17].

Here, we report the first reported case of a collisional ovarian tumor in a 14-year-old girl, composed of a combined (cystadenoma/borderline/cystadenocarcinoma) tumor and mature teratoma. In addition, we provide a brief review of the relevant literature.

## 2. Case Presentation

### 2.1. Patient Information

A 14-year-old girl was referred from the Community Health Center for abdominal swelling that had appeared three months earlier. Apart from the abdominal swelling, there were no other symptoms. Over the past six years, she had been treated by a pediatric gastroenterologist on several occasions for constipation and encopresis, which were treated with bisacodyl suppositories and macrogol oral sachets. The girl was born from her mother’s first pregnancy by cesarean section due to stasis in the birth canal, Apgar score of 10/10. In early childhood, she had febrile convulsions without the need for therapy. The mother was treated for ductal carcinoma in situ of the breast. The maternal grandmother had colon cancer, and the maternal grandfather had prostate cancer. The father is healthy, and throat cancer and a brain tumor have been described in the paternal grandparents. The girl’s younger brother is healthy.

### 2.2. Clinical Findings

Clinical examination by a pediatric surgeon revealed a distended abdomen without pain on palpation. The perianal area was normal, and urination and stool were regular. Gynecological examination revealed that the external genitalia were of normal color and structure, with a normal vestibule, denticular hymen, and normal vaginal depth. Menstrual cycles were regular, without pain.

### 2.3. Diagnostic Assessment

**Figure 1:**
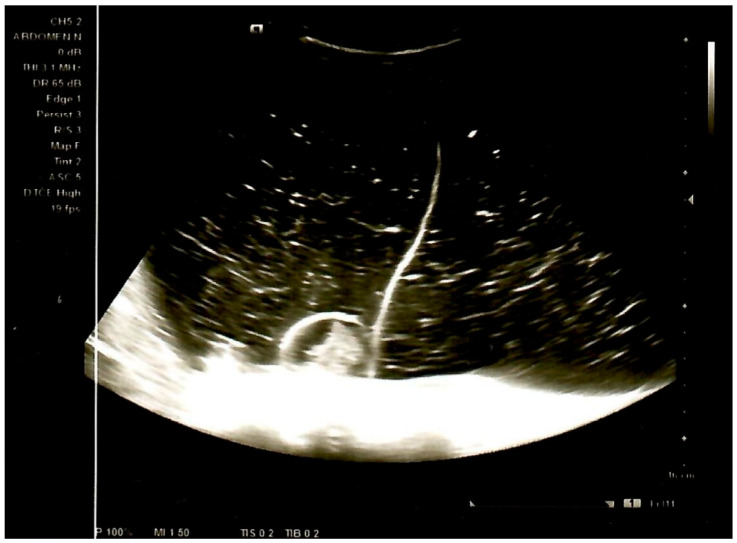
Abdominal US showed a septated multilocular cystic mass with internal dense contents and parts with solid components.

An urgent ultrasound (US) examination of the abdomen revealed an extensive cystic mass with multiple septa, filling the entire abdomen from the diaphragm to the pelvis, of open etiology, with a possible teratoma as a differential diagnosis. Ultrasound status of the liver, gallbladder, pancreas, spleen, and kidneys was normal. There was no free fluid in the abdomen (Figure 1).

**Figure 2:**
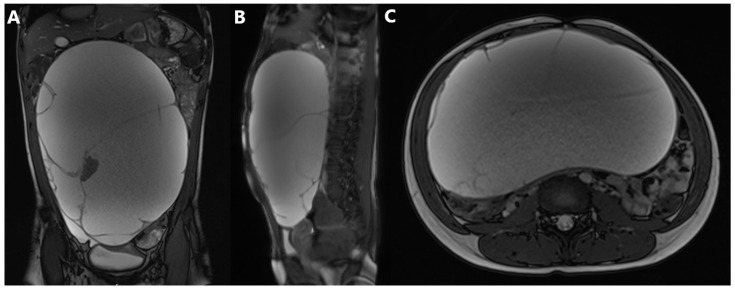
MRI of the abdomen in (**A**) coronal, (**B**) sagittal, and (**C**) transverse planes showing a tumor mass measuring 22cm × 13cm × 30 cm (LL × AP × CC).

Magnetic resonance imaging (MRI) of the abdomen, native and post-contrast, according to the standard protocol, showed an intraperitoneal mass, inseparable from the right ovary, measuring 22 cm × 13 cm × 30 cm (LL × AP × CC), mostly cystic, locally septate, with smaller solid components, primarily corresponding to a teratoma (Figure 2). The left ovary and uterus were normal. The pancreas was displaced dorsally, while the abdominal aorta was located left ventrolaterally along the vertebral bodies.

Normal acid-base, hematological, and biochemical levels were determined through extensive laboratory diagnostics. The urine test was normal. The levels of tumor markers were as follows: lactate dehydrogenase (LDH) 182 U/L, ferritin (FER) 19.6 μg/L, human chorionic gonadotropin (HCG) < 0.5 IU/L, neuron-specific enolase (NSE) 18.7 ng/mL, alpha-fetoprotein (AFP) 6 ng/mL, carcinoembryonic antigen (CEA) 1.7 μg/L, cancer antigen 125 (CA 125) 13.5 kIU/L, cancer antigen 19-9 (CA 19-9) 11.6 kIU/L, and cancer antigen 15-3 (CA 15-3) 9.7 kIU/L.

### 2.4. Therapeutic Intervention

**Figure 3:**
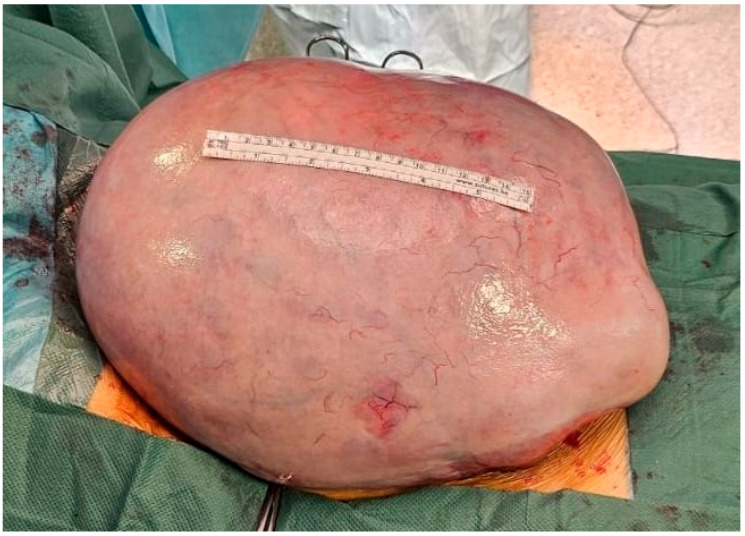
Luxated tumor mass from the abdomen after midline laparotomy. A 15 cm long measuring ruler on the tumor mass.

Not long after the diagnostic workup, surgical treatment was initiated. After midline laparotomy, the previously described tumor mass was encountered, which filled the entire abdomen. After the luxation of the mass from the abdomen, the starting point of the mass was verified in the area of the right ovary, which was completely penetrated by the tumor. Along with the extirpation of the tumor, an adnexectomy was performed (Figure 3). After extirpation, during which care was taken to avoid possible rupture and peritoneal dissemination of the contents, a cytological sample of the abdominal lavage and a biopsy of the omentum were taken. The abdominal cavity was thoroughly lavaged. The tumor mass was sent for pathohistological analysis. The introduction, course, and awakening from anesthesia, as well as the early postoperative recovery, were uneventful. The girl was discharged from the hospital on the seventh day.

### 2.5. Histopathological Analysis

**Figure 4:**
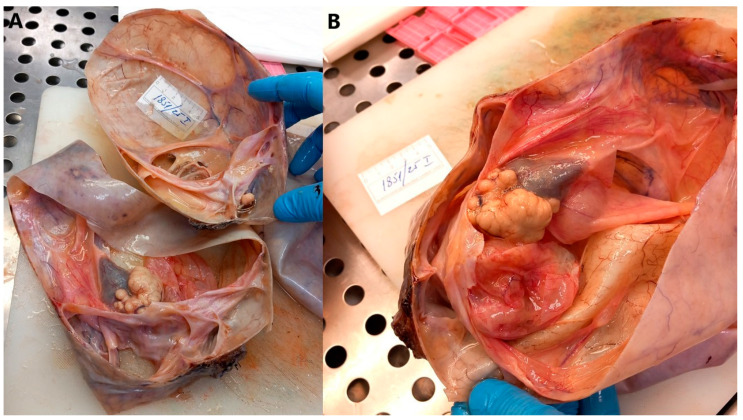
Multilocular cystic tumor with mucinous content and smooth external surface. (**A**) Grossly, a solid area 4 cm in diameter is visible on the internal surface of the cyst (left side of the image). Also, a white solid area 1 cm in diameter is present on the internal surface of the cyst (right side of the image). (**B**) Multilocular cystic tumor with a solid area on the internal surface of the cyst.

Macroscopically, a multilocular cyst measuring 28 cm × 22 cm × 14 cm was verified, with a wall thickness of up to 0.2 cm, a smooth outer surface on which a fallopian tube measuring 7 cm in length and 0.5 cm in diameter was found. In the lumen, several multilocular cysts were found, filled with yellowish fluid on the surface, and a solid area measuring 4 cm × 2 cm. At the edge, attached to the wall of the described cystic formation, a whitish area measuring up to 1 cm in diameter was found on the surface of which sebum and hair were found, macroscopically resembling a teratoma (Figure 4).

**Figure 5:**
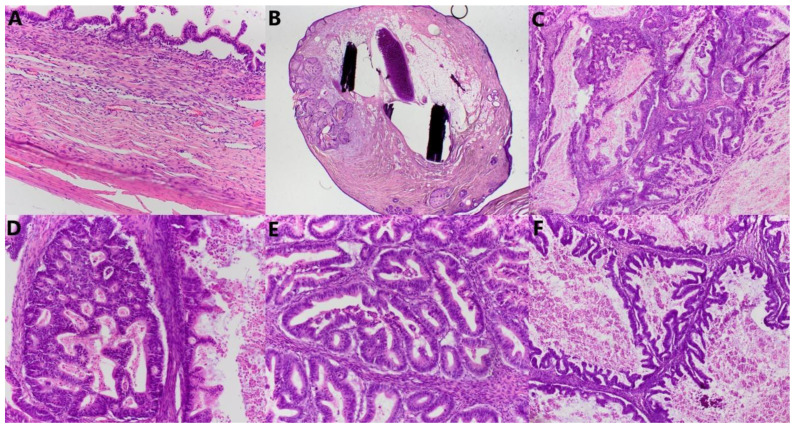
Microscopic sections of the tumors. (**A**) Fibrous wall of the tumor lined by one layer of bland mucinous epithelium (HE × 200), (**B**) mature teratoma composed of fibrous tissue, mature fatty tissue, cartilage, and sebaceous glands lined by epidermis (HE × 20), (**C**) complex architecture composed of glandular and cribriform formations with atypical epithelium (HE × 100), (**D**) cribriform formation with atypical, mitotically active epithelium (HE × 200), (**E**) crowded glandular formations with atypical, mitotically active epithelium (HE × 200), (**F**) complex architecture with tufting and villus formation (HE × 100).

**Figure 6:**
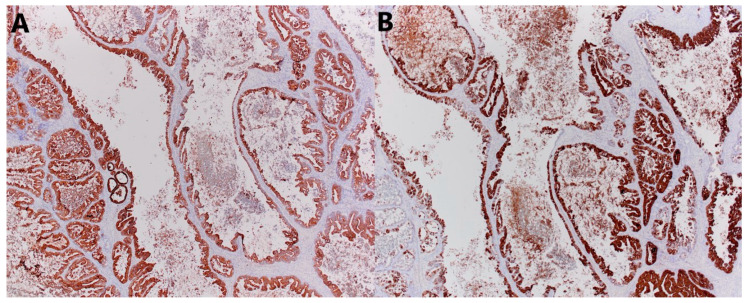
Microscopic sections of tumors. Positive immunohistochemical reaction for (**A**) CK7 and (**B**) CK20 (×40).

**Figure 7:**
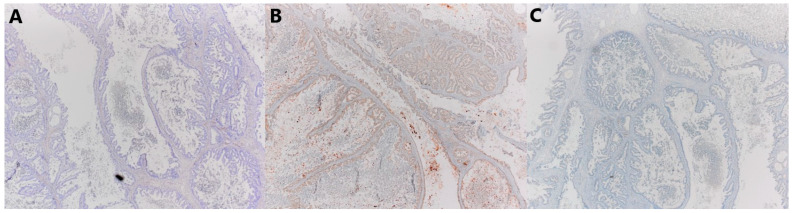
Microscopic sections of tumors. Negative immunohistochemical reaction for (**A**) ER, (**B**) PAX8, and (**C**) SATB2 (×40).

Microscopically, the smaller described area is a mature teratoma made up of adipose tissue, connective tissue, and mature cartilage, and a cyst lined with multilayered squamous epithelium, under which in the underlying connective tissue are the structures of the skin adnexa. The larger cystic formation is partly made up of a thin connective wall lined with a single layer of cylindrical epithelium (intestinal type), with nuclei located basally, without major atypia and mitoses. In part, there is a complex architecture, branched papillary and cribriform structures lined with the same type of epithelium, with milder cell atypia and so-called tufting. In up to 10% of tumors, there are also partitioned and compact glandular and cribriform formations lined with atypical epithelium and embedded in minimal stroma, with sharp borders, which corresponds to foci of adenocarcinoma, an expansive (confluent) type of growth. Focal remnants of ovarian tissue are seen at the edges (Figure 5). Immunohistochemically, cells were diffusely positive for CK7, mostly for CK20, and CDX2 (Figure 6), and negative for ER, PR, PAX8, SATB2, and CD68 (Figure 7).

Macroscopic and histological examination showed that the mass was completely removed. The resection margins were free of tumor. The omentum tissue was unremarkable. No tumor cells were found in the lavage of the abdomen.

In conclusion, the histological and immunohistochemical analysis corresponds to mucinous cystadenocarcinoma, characterized by a transition pattern from benign and borderline components to an adenocarcinoma component with a confluent growth pattern, which accounts for up to 10% of tumors, FIGO stage IA, TNM pT1a of the eighth edition of the TNM classification. In addition to the mentioned tumor, there was a smaller mature teratoma.

### 2.6. Follow-Up and Outcomes

**Figure 8:**
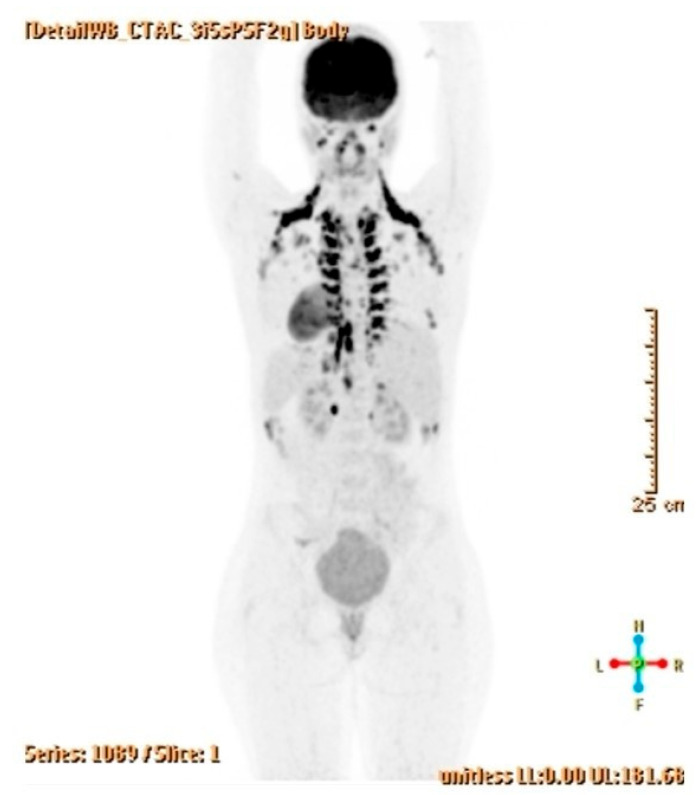
A normal PET-CT scan three months after surgery.

Postoperatively, a six-month follow-up to date has not revealed any recurrence or postoperative complications. The control US, MRI, and PET-CT scans were completely normal, as were the levels of tumor markers (Figure 8). Genome sequencing is underway, based on which genetic counseling will be conducted. Close follow-up of the patient continues.

## 3. Discussion

We presented the first case of a successfully diagnosed and treated ovarian collision tumor composed of a combined (cystadenoma/borderline/cystadenocarcinoma) tumor and mature teratoma in a 14-year-old girl.

To date, collision tumors have been reported in various organs, predominantly in the digestive and endocrine organs, while they are much rarer in the ovary [15,18,19,20]. They are quite challenging because the missed diagnosis of the second component of the collision tumor can lead the treatment and the outcome in an unwanted direction [21]. The typical collision patterns observed in ovarian collision tumors are the “back-to-back” and “nested tumor” patterns, where the larger tumor typically represents the epithelial tumor, while the smaller tumor may indicate a germ cell or sex cord-stromal tumor, as was observed in our case [15]. Collision tumors should not be confused with combined or composite tumors, which contain a mixture of different histological components originating from the same stem cell type, respectively, a mixture of histologically distinct neoplasms without a defined interface [22,23]. In our case, we also had an example of a combined tumor (cystadenoma/borderline tumor/cystadenocarcinoma) where both benign and malignant components were present at the same time, with varying degrees of cellular atypia. Based on the above, we can say that we had both a combined and a collision tumor in the same ovary in the patient, which has not been recorded to date.

As in our case, almost all previously described ovarian collision tumors to date have been various combinations of epithelial tumors, germ cell tumors, and sex cord-stromal tumors (most often mucinous cystadenoma/cystadenocarcinoma and teratoma), with a mean age at presentation of 40, 39, and 37 years, respectively. According to studies conducted to date, clinical manifestations are nonspecific, but patients usually complain of intermittent abdominal pain and bloating, and frequent urination [15,24,25]. The patient in our case did not have any complaints except abdominal distension, which she did not associate with anything pathological. Although elevated levels were not observed in our case, elevated CA-125 levels are the most common abnormality, suggesting an epithelial component of the ovarian collision tumor [26,27]. The absence of elevated CA-125 in a collision ovarian tumor consisting of a combined tumor and mature teratoma likely results from the tumor’s composite nature—specifically, the contribution of the mature teratoma component, which does not produce CA-125, and potentially a limited or less active cystadenocarcinoma component. This underscores that CA-125 levels are not universally elevated in all ovarian tumors, especially when benign or mature components predominate [12]. Certain ovarian tumor subtypes, such as some germ cell tumors or borderline tumors, may not significantly elevate CA-125 levels and may also lack ER/PR expression. The absence of elevated CA-125 in the context of ER/PR negativity might suggest a less advanced or less biologically aggressive tumor, but clinical correlation, imaging, and pathological assessment are essential for accurate staging and management [28]. Although the radiologists in our case primarily attributed the tumor to a teratoma, the diagnosis of a collision ovarian tumor should be considered when two or three types of typical imaging findings of different tumors are present in the same ovary, especially when one tumor lies within or on the wall of another tumor, or when the overall imaging findings of a suspected teratoma do not correspond to its typical radiological features, or when patients present with confusing clinical manifestations, which could only be explained by a mixture of different tumor components [15,29]. Given the patient’s age, preserving ovarian tissue on the affected side would have been the preferred surgical choice, but since the tumor had extensively infiltrated the ovary with an invasive appearance, it was decided to perform a unilateral salpingo-oophorectomy while avoiding rupturing the tumor mass during surgery to prevent leakage, which was achieved. Without the removal of the involved ovary, complete surgical staging and cytoreduction might not have been achieved, which are critical for optimal oncologic outcomes. Persistence of tumor tissue could negatively influence the patient’s prognosis, increasing the likelihood of recurrence or progression. If the tumor progressed, a more extensive second surgery might have been necessary later, which could be more complicated and carry increased risks [30]. While ovarian preservation is often desirable in young patients to maintain fertility and hormonal function, this must be balanced against oncologic safety [31].

Differentiating primary from secondary mucinous ovarian tumors using immunohistochemistry is an important diagnostic step because it influences treatment and prognosis [32,33]. Looking at the results of immunohistochemical analysis, which determined CK7 positivity with focal CK20 and CDX2 positivity, correlating them with the clinical presentation and radiological findings both preoperatively and during follow-up, everything indicates that this is a primary, and not a secondary (metastatic) mucinous ovarian tumor. Particular care should be taken to avoid misdiagnosing ovarian metastases as primary ovarian tumors due to pathological features that may mimic primary ovarian tumors. In most such cases, clinical and radiological examination reveals a primary tumor elsewhere, but care must be taken to ensure that it does not become apparent later during follow-up. The key to distinguishing primary mucinous ovarian tumors from metastatic occult gastrointestinal tumors during follow-up involves a combination of immunohistochemistry, morphological assessment, thorough initial and ongoing imaging (US, CT, MRI, endoscopy), and clinical evaluation. The absence of detectable primary lesions in the gastrointestinal system, along with consistent histopathological and immunohistochemical findings, supports the conclusion that the tumor is primarily localized in the ovary [33,34]. Early detection and appropriate treatment generally lead to excellent outcomes with ovarian collision tumors composed of cystadenocarcinoma and mature teratoma. Regular follow-up with tumor markers and imaging is essential for early detection of recurrence [35,36]. The current six-month follow-up period for tumors containing a malignant component is relatively short. The follow-up plan must be multidisciplinary, by an oncologist, radiologist, gynecologist, and surgeon. Ultrasound and MRI scans will be performed every three months during the first year, every six months during the second year, and then annually after two years of follow-up if the condition is stable, for a total of at least five years. Serum tumor markers will also be determined in parallel with regular clinical examinations. Potential impacts on ovarian reserve and future fertility will also be discussed by the gynecologist. Genetic counseling is planned after genome sequencing, which will include a detailed pedigree assessment, discussion of potential hereditary syndromes, and family counseling to guide management and surveillance.

## 4. Conclusions

We have presented the first case of a pediatric ovarian collision tumor that includes both malignant epithelial components and benign teratoma tissue. The recognition of such a unique tumor emphasizes the importance of thorough radiological and histopathological evaluation of pediatric ovarian masses, guiding appropriate surgical and oncological treatment strategies. As the first documented case, this case provides valuable insights into pediatric ovarian neoplasms, guiding future diagnostic and therapeutic approaches.

## Data Availability

Data on the patient’s follow-up are available upon request from the corresponding author.

## Abbreviations

The following abbreviations are used in this manuscript:

LL	Latero-lateral
AP	Antero-posterior
CC	Cranio-caudal
US	Ultrasound
MRI	Magnetic resonance imaging
PET-CT	Positron emission tomography–computed tomography
LDH	Lactate dehydrogenase
FER	Ferritin
HCG	Human chorionic gonadotropin
NSE	Neuron-specific enolase
AFP	Alpha-fetoprotein
CEA	Carcinoembryonic antigen
CA	Cancer antigen
FIGO	International Federation of Gynecology and Obstetrics
